# Digital Prediction of the Purchase Price of Fresh Tea Leaves of Enshi Yulu Based on Near-Infrared Spectroscopy Combined with Multivariate Analysis

**DOI:** 10.3390/foods12193592

**Published:** 2023-09-27

**Authors:** Shengpeng Wang, Lin Feng, Panpan Liu, Anhui Gui, Jing Teng, Fei Ye, Xueping Wang, Jinjin Xue, Shiwei Gao, Pengcheng Zheng

**Affiliations:** 1 Key Laboratory of Tea Resources Comprehensive Utilization, Ministry of Agriculture and Rural Affairs, Institute of Fruit and Tea, Hubei Academy of Agricultural Sciences, Wuhan 430064, China; wwsspp0426@163.com (S.W.);; 2 Hubei Tea Engineering and Technology Research Centre, Wuhan 430064, China

**Keywords:** fresh tea leaves, price, near-infrared spectroscopy, synergy interval partial least squares, back propagation-artificial neural network, transfer function

## Abstract

In this study, near-infrared spectroscopy (NIRS) combined with a variety of chemometrics methods was used to establish a fast and non-destructive prediction model for the purchase price of fresh tea leaves. Firstly, a paired *t*-test was conducted on the quality index (QI) of seven quality grade fresh tea samples, all of which showed statistical significance (*p* < 0.05). Further, there was a good linear relationship between the QI, quality grades, and purchase price of fresh tea samples, with the determination coefficient being greater than 0.99. Then, the original near-infrared spectra of fresh tea samples were obtained and preprocessed, with the combination (standard normal variable (SNV) + second derivative (SD)) as the optimal preprocessing method. Four spectral intervals closely related to fresh tea prices were screened using the synergy interval partial least squares (si-PLS), namely 4377.62 cm^−1^–4751.74 cm^−1^, 4755.63 cm^−1^–5129.75 cm^−1^, 6262.70 cm^−1^–6633.93 cm^−1^, and 7386 cm^−1^–7756.32 cm^−1^, respectively. The genetic algorithm (GA) was applied to accurately extract 70 and 33 feature spectral data points from the whole denoised spectral data (DSD) and the four characteristic spectral intervals data (FSD), respectively. Principal component analysis (PCA) was applied, respectively, on the data points selected, and the cumulative contribution rates of the first three PCs were 99.856% and 99.852%. Finally, the back propagation artificial neural (BP-ANN) model with a 3-5-1 structure was calibrated with the first three PCs. When the transfer function was logistic, the best results were obtained (R_p_^2^ = 0.985, RMSEP = 6.732 RMB/kg) by 33 feature spectral data points. The detection effect of the best BP-ANN model by 14 external samples were R^2^ = 0.987 and RMSEP = 6.670 RMB/kg. The results of this study have achieved real-time, non-destructive, and accurate evaluation and digital display of purchase prices of fresh tea samples by using NIRS technology.

## 1. Introduction

Enshi Yulu is a famous historical tea produced in Enshi City, Hubei Province. It is the only steamed green tea and is a national geographical indication protection product in China [1]. Its brand reputation is well-known at home and abroad, and is often used to entertain foreign guests in state events. Enshi Yulu Nature Reserve is located in the range of 450–850 m above sea level. The climate is warm and humid all year round, and the clouds and mists around the day and night produce more diffuse light and short-wave ultraviolet light, making the fresh tea buds and leaves tenderer, and the contents of protein, amino acid and alkaloid are very rich, which lays a solid material foundation for forming the excellent quality of Enshi Yulu.

As is known to all, the quality of fresh tea leaves is the basis of the tea quality [2]. For example, before Enshi Yulu is processed, there are clear regulations on the quality of fresh tea leaves [3]; for example, the special grade one Enshi Yulu requires fresh tea leaves of a single bud to exceed 95% so that the raw materials are fresh and uniform, without red bud leaves, purple bud leaves, disease and insect bud leaves, and rain leaves. Due to the high requirements on the quality of fresh tea leaves, the purchase price of Enshi Yulu fresh tea leaves is higher than that of ordinary fresh tea leaves.

Generally speaking, tea gardens are usually planted and managed by tea farmers, and tea processing factories are responsible for purchasing and processing fresh tea leaves. When purchasing, the purchasing personnel usually determine the quality grade of fresh tea leaves based on their own sense organs, such as smell, vision, and touch, and personal experience, and then give the corresponding purchase price. For example, Zhang Jun [4] has established a relationship between the quality and price of jasmine tea. However, the sensitivity of human sense organs is easily affected by their own work experience, physiological conditions at that time, and external conditions (such as the surrounding environment, weather, temperature, and humidity), and has greater subjectivity, so most of the time the purchaser and the tea farmer could not reach an agreement on the determination of the purchase price of fresh tea leaves, resulting in many conflicts. To create an effective method to weaken the limitations of sensory quality analysis and compensate for the shortcomings (e.g., subjectivity, unpredictability, and inconsistency) of sensory evaluation, researchers have been devoted to developing various instrumental analytical techniques to evaluate the quality of fresh tea leaves based on their physical and chemical profiles, such as liquid chromatography-mass spectrometry [5], gas chromatography–mass spectrometry [6], and high-performance liquid chromatography [7]. Then, the quality index (QI) of fresh tea leaves was computed by selecting proprietary ingredients [8] in order to evaluate the purchase price based on quality. Although the chemical method has high detection accuracy, the process is extremely cumbersome and requires many chemical reagents. In addition, the detection process is time-consuming and laborious, and cannot have timeliness. It is still unable to achieve the rapid evaluation of the purchase price of fresh tea leaves. Therefore, in order to effectively alleviate the distrust between tea factories and tea farmers and increase their mutual trust between each other, it is urgent to establish an objective, fair, and rapid method for digitally evaluating the purchase price of Enshi Yulu fresh tea leaves.

Near-infrared spectroscopy (NIRS), an electromagnetic wave with a wavelength in the range of 780–2526 nm, mainly reflecting the X–H chemical bond, has the advantages of rapid and non-destructive analysis, and now has been widely used in agriculture [9,10,11], the petrochemical industry, the textile industry, and the pharmaceutical industry [12,13]. NIRS combined with CARS-PLS and si-PLS methods, has been broadly used to predict the amounts of polyphenols, caffeine [14], and other components in tea [15], assess the quality of fresh tea leaves using QI values [16], and discriminate the tea varieties [17]. However, at present, there are few reports on the application of near-infrared spectroscopy technology to evaluate the purchase price of fresh tea leaves, and further research is needed.

In this paper, fresh tea leaves of the Entaizao tea varieties in the Enshi Yulu Nature Reserve were used as the research objects, the quality index (QI) of different quality grades (QG) were calculated, and the corresponding relationship between QI, QG, and the purchase price was clarified. Then, NIRS were obtained, and the spectral noise information was removed by various pre-processing methods. The characteristic spectral data was extracted by using the synergy interval partial least squares (si-PLS) method and genetic algorithm (GA), then the principal component analysis (PCA) method was applied to compress and extract the above characteristic spectral information, and finally, the back-propagation artificial neural network (BP-ANN) method combined with three transfer functions was used to establish a NIRS digital model of the purchase price. The actual application effect of the model was tested using external samples. This study can provide a convenient new method for the rapid, non-destructive, objective, and digital evaluation of the purchase price of Enshi Yulu fresh tea leaves, striving to overcome subjective factors and laying a solid scientific foundation for the next step of developing a portable near-infrared spectrometer for the purchase price of fresh tea leaves.

## 2. Materials and Methods

### 2.1. Samples and Classification of Fresh Tea Leaves

Samples of Entaizao tea variety fresh tea leaves were picked from March to May 2022. The sample standard was one bud, one bud and the first leaf, one bud and the first two leaves, and one bud and the first three leaves. Then, seven quality grades of fresh tea processing samples were mixed with the above fresh tea leaves [3] (Table 1), (grade 1 samples have the best quality, while grade 7 samples have the worst quality). Each grade had 8 samples and each sample were approximately 100 g. In order to ensure the fairness of fresh leaf prices, in this study the price was set by a team of three people, including one fresh leaf purchaser, one tea expert, and one tea farmer. The prices of fresh leaf tea are set based on the quality of fresh leaves. The quality of fresh leaves is closely related to their tenderness, integrity, uniformity, and purity. The better the tenderness of fresh leaves, the fresher they are, which is beneficial for kneading and shaping during processing. The integrity of fresh leaves is good, indicating that the buds and leaves of fresh leaves are not separated, and the finished tea has less broken tea. The high uniformity of fresh leaves indicates that the picking standards for fresh leaves are consistent, making it easy to use the same processing parameters and improving the utilization rate of fresh leaves. The purity of fresh leaves is good, indicating that there are fewer impurities in the fresh leaves, such as grass leaves and other leaves. Therefore, the better the quality of fresh leaves, the higher the price of the fresh leaves. In addition, considering the market demand factor and local price levels when purchasing fresh leaves, the average purchase price of fresh leaves from grade 1 to grade 7 ranges from 30 RMB/kg to 220 RMB/kg in this research. According to the different quality grades, of the 56 samples, 42 were selected for the calibration set model and 14 were used for validation, with a ratio of 3:1. Additionally, 14 samples purchased from the local market were used to test the effectiveness of the price calibration model.

### 2.2. Spectral Collection

**Figure 1:**
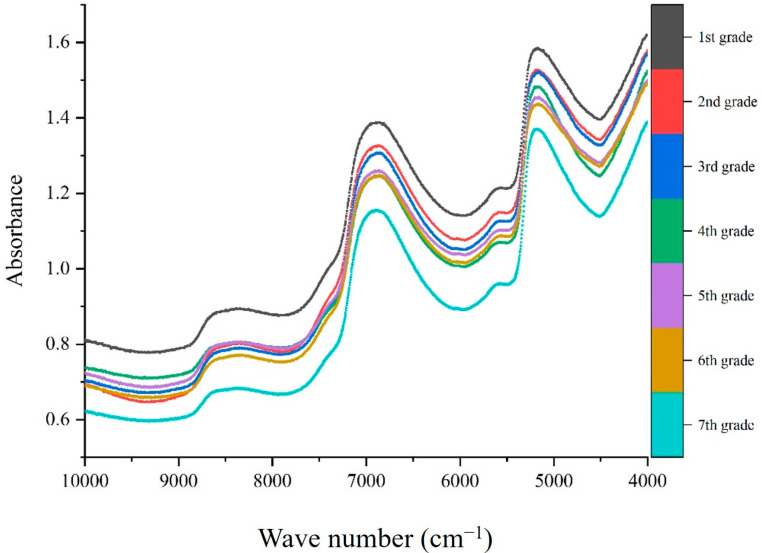
Near-infrared spectroscopy of seven quality grades fresh tea samples.

Near-infrared spectroscopy (NIRS) data were acquired using a Thermo Antaris II Fourier transform (FT) NIR spectrometer (Thermofisher Scientific, Waltham, MA, USA) in reflectance mode, equipped with an InGaAs detector and an integrating sphere accessory. To obtain the spectral data, 10 g of the fresh tea samples were placed in a sample cup that rotated 360° during scanning. The spectral range was between 10,000 cm^−1^ and 4000 cm^−1^, with 3.857 cm^−1^ intervals. Each sample was scanned three times, and the average spectrum of the three scans was used for subsequent analysis (Figure 1).

### 2.3. Spectral Data Analysis

Due to the distance between adjacent spectral data points being 3.857 cm^−1^, the near-infrared spectrum of each sample contains 1557 pairs (x, y) of data points (x as spectral data points, y as absorbance) analyzed by TQ Analyst 9.4.45 software (Thermofisher Scientific, Waltham, MA, USA). The data point pairs were saved in Excel sheets. Then, the PLS model was built to select the best pretreatment method by using OPUS 7.0 software (Bruker Optik GmbH., Saarbrucken, Germany). The feature spectral intervals were selected to build price NIRS models by using the synergy interval partial least squares (si-PLS) method on the Matlab 2012a software platform (MathWorks, Natick, MA, USA).

Before modeling, in order to effectively eliminate extraneous background and noise information and enhance model performance, various spectral preprocessing techniques were employed, including spectral free preprocessing (none), standard normal variable (SNV), first derivative (FD), second derivative (SD), multiple scatter correction (MSC), and their combined methods, to remove noise from the original spectra [18]. After comparing the results, the optimal preprocessing method was determined.

### 2.4. Modeling Methods

#### 2.4.1. Synergy Interval Partial Least Squares (si-PLS) Method

The si-PLS method can divide the whole spectral data set into a number of intervals (10–25), and all possible PLS models combinations for two, three, or four intervals are calculated [19]. The spectral regions most relevant to the price are selected according to the root mean square error of cross-validation (RMSECV) in the calibration set. When the si-PLS model has the lowest RMSECV, the modelled spectral intervals are the selected feature spectral intervals, which contain the NIR information specific to the price of fresh tea samples.

The RMSECV was calculated as follows:

(1) $$\mathrm{RMSECV}=\sqrt{\frac{\sum_{i=1}^{n}{\left(y_{i}^{\prime }-y_{i}\right)}^{2}}{n}}$$

where *n* is the number of samples in the calibration set, *y_i_* is the true value for sample *i*, and *y_i_*′ is the theoretical value for sample *i* predicted from the calibration set.

#### 2.4.2. Genetic Algorithm (GA)

The GA refers to natural selection and genetic mechanism in the biological world [20]. It uses operators such as selection, exchange, and mutation. With continuous genetic iteration, the variables with better objective function values are retained, and the poor variables are eliminated, and lastly, the optimal results are achieved. In this paper, the GA is applied to obtain the optimal NIRS data points, when RMSECV is at its minimum.

#### 2.4.3. Principal Component Analysis (PCA) and Backpropagation Artificial Neural Network Method (BP-ANN)

PCA [21] was performed on the best spectral intervals and data points obtained by the si-PLS method and GA method, respectively. It is the compression and extraction of spectral information to obtain the contribution rates of each principal component, which is used to establish a BP-ANN model.

The BP-ANN [22] has emerged as a research hotspot in the field of artificial intelligence in recent years. It abstracts the neural network of the human brain from the perspective of information processing, forms different networks according to different connection modes, and is composed of a large number of neurons connected with each other. Each node represents a specific transfer function. By establishing the connection between input data and output data, a prediction model can be established. The results were evaluated based on the coefficient of determination of cross-validation (Rc^2^), coefficient of determination of prediction (Rp^2^), root mean square error of cross-validation (RMSECV), and root mean square error of prediction (RMSEP). A higher R^2^ and a lower RMSEP indicate better prediction performance. The equations used to calculate RMSEP and R^2^ are provided.

The RMSEP was calculated as follows:

(2) $$\mathrm{RMSEP}=\sqrt{\frac{\sum_{i=1}^{n}{\left(y_{i}-y_{i}^{\prime }\right)}^{2}}{n}}$$

where *n* is the number of samples in the prediction set, *y_i_* is the true value of sample *i* and *y_i_*′ is the predicted value of sample *i* in the prediction set.

The R^2^ was calculated as follows:

(3) $$R^{2}=1-\frac{\sum_{i=1}^{n}{\left(y_{i}^{\prime }-y_{i}\right)}^{2}}{\sum_{i=1}^{n}{\left(y_{i}^{i}-\bar{y}\right)}^{2}}$$

where *y_i_* and *y_i_*′ are the true value and predicted value of sample *i*, respectively, and $\bar{y}$ is the average true value of all samples.

## 3. Results and Discussion

### 3.1. The Relationships between Quality Grade, Quality Index, and Purchase Price of Fresh Tea Samples

According to the formula for calculating the quality index (QI) (QI = (humidity content × total nitrogen content) ÷ crude fiber content) [23], the QI values of seven quality grades of fresh tea samples were obtained and a paired *t*-test was conducted. The results are shown in Table 2.

Table 2 shows that the value of t (1v2) was the smallest at 3.25, but it was still greater than the critical value of 2.365 (t_0.05_ (7) = 2.365). Therefore, there was significant statistical significance (*p* < 0.05) between the first and second QG of fresh tea samples. The t-values among the other QG were all greater than the critical value of 3.499 (t_0.01_ (7) = 3.499), so there was extremely significant statistical significance (*p* < 0.01) among the other QG of fresh tea samples. Therefore, Table 2 confirms the correctness of the classification of fresh tea samples, laying a foundation for the next research on the purchase prices of fresh tea samples of different quality grades.

**Figure 2:**
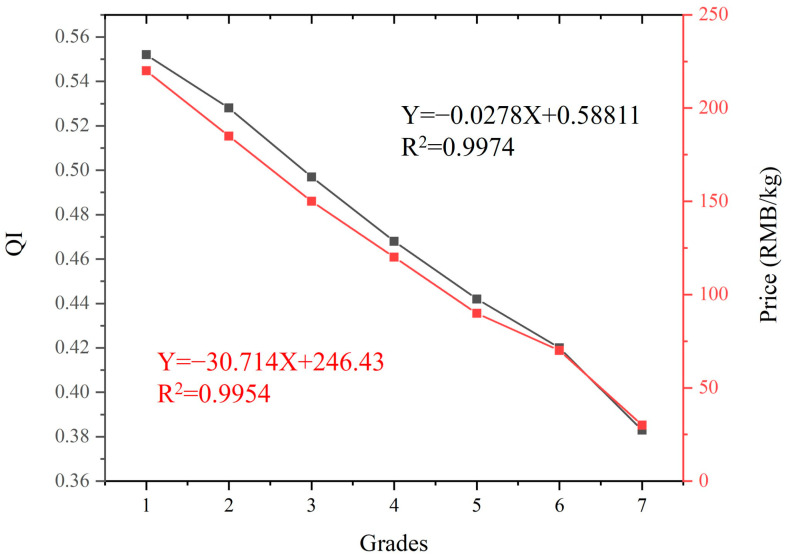
Relationships between average QI values, average purchase prices, and seven QGs of fresh tea samples.

Figure 2 shows that as the quality of fresh tea samples gradually decreased from grade 1 to grade 7, their QI values also showed a gradual downward trend. The QI of grade 1 fresh tea leaves was the highest at 0.552, while the QI of grade 7 fresh tea leaves was the lowest at 0.383. From grade 1 to grade 7, the QI values decrease by 30.6%, indicating that the quality of the mixed fresh tea samples was very reasonable, and there was a good linear relationship between the quality grades and the quality index, with an excellent correlation, an R^2^ of 0.9974.

Figure 2 also shows that as the quality of fresh tea samples gradually decreased, their average purchase prices decreased rapidly from the highest 220 RMB/kg for grade 1 to 30 RMB/kg for grade 7. By fitting the relationship between the quality grade and the purchase price of fresh tea samples, the correlation was excellent, and the R^2^ was as high as 0.9954. Therefore, there was a good linear relationship between the quality grade and the purchase price of fresh tea samples.

**Figure 3:**
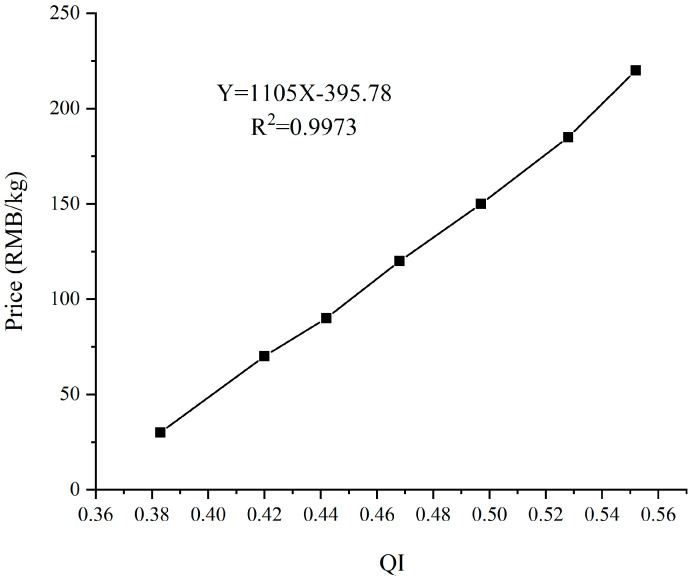
The relationship between the average QI values and the average prices of seven grade samples.

Figure 3 shows that by fitting the linear relationship between the average QI values and their corresponding average purchase prices of seven grades of fresh tea samples, it was found that there was also an excellent linear relationship between the two factors, with an R^2^ as high as 0.9973. Therefore, based on the quality of fresh tea samples, it will be a completely feasible and convenient method to quickly and non-destructively predict the purchase price of fresh tea leaves by using NIRS technology.

### 3.2. Comparison of Pre-Processing Methods for Spectral Data

**Figure 4:**
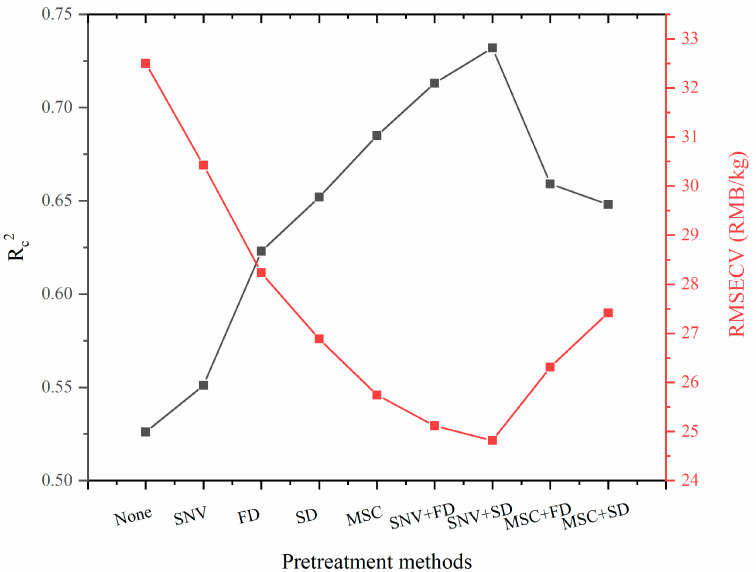
The results of purchase price PLS models by using different pretreatment methods.

Figure 1 shows that the spectra exhibit multiple absorption peaks in the long wave band (4000–7000 cm^−1^), primarily due to the presence of water −OH [25] and various components of varying quality in the NIRS absorption information of fresh tea samples. Prior to model building, nine spectral preprocessing methods were employed to pretreat the NIR spectra of fresh tea samples with varying quality prices. Subsequently, PLS was utilized to construct NIRS models. The performance of the models was evaluated using RMSECV and Rc^2^, with a higher Rc^2^ and lower RMSECV indicating better pretreatment methods. The results of all the pre-treatment models are presented in Figure 4.

In Figure 4, among the nine models, the NIRS models built with the original spectra yielded the worst results (Rc^2^ = 0.526, RMSECV = 32.501 RMB/kg). The models with a single preprocessing method, specifically the MSC pretreatment method, showed better results (Rc^2^ = 0.685, RMSECV = 25.742 RMB/kg), but the prediction results were still inferior to those obtained with combined pre-treatment methods. The NIRS model built using the (SNV+SD) combined method produced the best results (Rc^2^ = 0.732, RMSECV = 24.817 RMB/kg), representing a 39.16% increase in Rc^2^ and a 23.64% decrease in RMSECV compared to the original spectra NIR model. Therefore, it is crucial to pretreat the original spectra before building NIRS models, which is consistent with previous findings [26]. In this study, the best spectral pretreatment method was the combination of (SNV + SD), and the Rc^2^ and RMSECV of the best calibration model built using the PLS method were 0.732 and 24.817 RMB/kg, respectively. This is because the SNV pre-processing method is used to correct spectral errors caused by scattering between samples; moreover, each spectrum is individually corrected separately. Its correction ability is superior to the MSC method. A derivative pre-treatment method can eliminate spectral baseline drift, enhance spectral band characteristics, and overcome spectral band overlap. Because there is less information directly reflecting the purchase price in NIRS, the application of a second derivative spectral pre-treatment method can highlight the NIRS information reflecting the purchase price. Therefore, the best spectral pre-treatment method obtained in this experiment was the combined method of (SNV + SD) [27]. However, the performance was still unsatisfactory, and there is still ample room for improvement in the results.

### 3.3. Results of si-PLS Model

As seen from Table 3, all spectra were divided into 10–25 spectral sub-intervals. Along with the numbers of the spectral intervals gradually increasing, the RMSECV of the si-PLS models showed a trend of gradually decreasing and then slowly increasing, but the PLS factors changed were not significant, with a range from 7 to 10. When the number of sub-regions was 16, and the factor number was 8, the results of the calibration model had the best performances, meaning the RMSECV value was the least, 15.340 RMB/kg. The selected spectral regions were the four regions of [2 3 7 10], and the corresponding spectral wavelengths were 4377.62 cm^−1^–4751.74 cm^−1^, 4755.63 cm^−1^–5129.75 cm^−1^, 6262.70 cm^−1^–6633.93 cm^−1^, and 7386 cm^−1^–7756.32 cm^−1^, respectively. The R_c_^2^ of the calibration model was 0.783. When the prediction samples were used to test the NIRS calibration model, the R_p_^2^ and RMSEP were 0.746 and 17.252 RMB/kg, respectively. Although the data information of the characteristic spectral range accounted for 25% of the all-spectral data information, the si-PLS prediction results were better than that PLS model. But, in practical applications, the accuracy of the si-PLS model still needs to improve further. Therefore, nonlinear methods were applied to establish a prediction model for the purchase prices of fresh tea leaves.

### 3.4. Results of BP-ANN Model

#### 3.4.1. Accurately Screening of Characteristic Spectral Data Points Using GA

**Figure 5:**
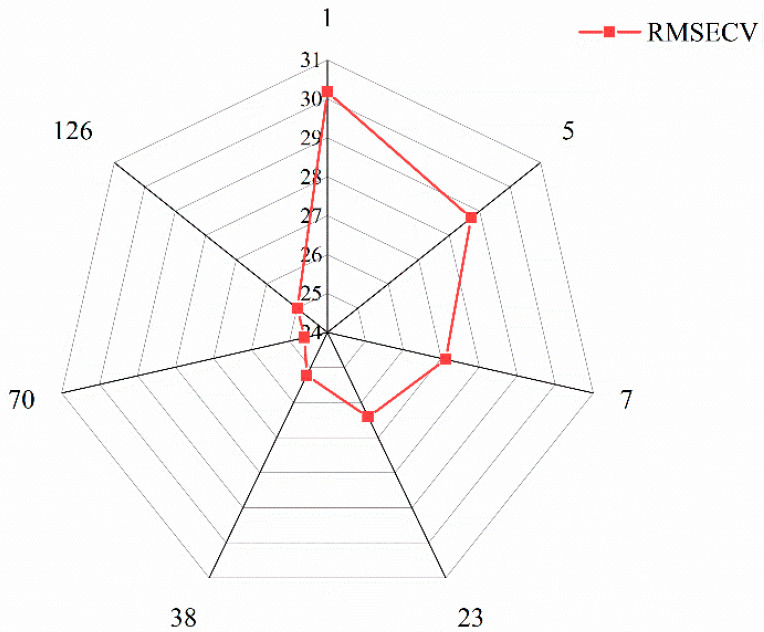
Correspondence between RMSECV and the best spectral data points of DSD.

**Figure 6:**
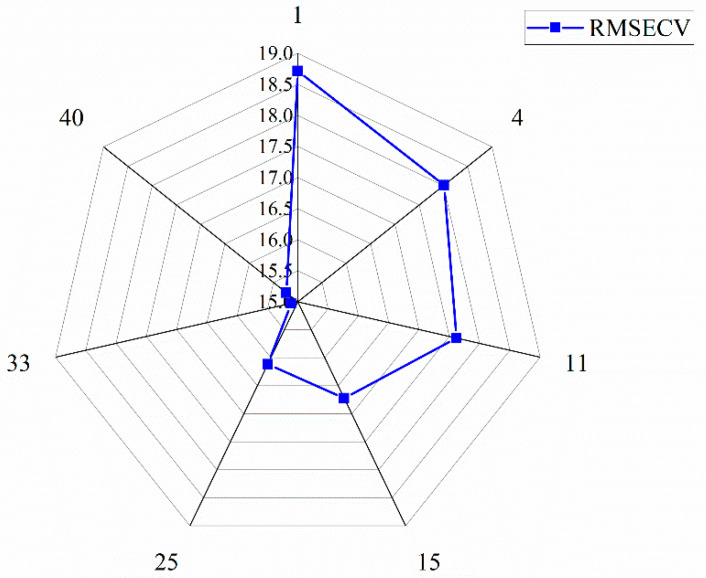
Correspondence between RMSECV and the best spectral data points of FSD.

In order to further improve the NIRS model prediction accuracy of purchase prices, the GA was applied to accurately extract spectral data points that reflect the purchase prices from whole denoised spectral data (DSD) and the four characteristic spectral intervals data (FSD) selected by si-PLS method, respectively. The RMSECV and the corresponding filtered feature spectral data points are shown in Figure 5 and Figure 6.

Figure 5 and Figure 6 show that, in the process of applying GA to extract feature spectral data points further accurately, whether for the DSD and FSD, as the feature spectral data points gradually increased, the RMSECV value showed a trend of rapidly decreasing to the minimum values and then gradually increasing. Among them, when the minimum RMSECV obtained 24.602 RMB/kg, 70 optimal spectral data points were extracted for DSD. Also, when the minimum RMSECV obtained 15.106 RMB/kg, 33 optimal data points were extracted for FSD. Comparing the results of Figure 4, the proportion of spectral data points (70 data points) extracted using the GA method to all spectral data points (1557 data points) was only 4.62%, but the RMSECV value was lower than the full wavelength PLS model (24.817 RMB/kg), indicating a slight improvement in prediction accuracy. Similarly, comparing the results of Figure 6 and Table 3, a total of 390 spectral data points were found in the four selected feature spectral intervals in Table 3. After further extraction, a total of 33 feature spectral data points were obtained, accounting for only 8.21% of the FSD. However, the RMSECV value (15.106 RMB/kg) was lower than the si-PLS model (RMSECV = 15.340 RMB/kg), indicating a slight improvement in the model’s prediction accuracy. From the above results, it can be further concluded that the GA had better spectral information extraction ability, which not only has eliminated noise information unrelated to purchase prices, but has also reduced the amount of data input to the model, which was very conducive to improving the prediction ability [28]. The extracted feature spectral data points are shown in Table 4.

From Table 4, it can be concluded that the characteristic spectral data points of DSD had a distribution within the full wavelength range, indicating that the data points can better represent the DSD information. Among the feature spectral data points extracted from FSD, a total of 23 feature spectral data points were extracted in the range of 4377.62 cm^−1^–5129.75 cm^−1^, 9 feature spectral data points were extracted in the range of 6262.70 cm^−1^–6633.93 cm^−1^, while only 1 feature spectral data point was extracted in the range of 7386 cm^−1^–7756.32 cm^−1^. This was because the near-infrared spectral information of fresh tea leaves was mainly reflected in the long wavelength range, which contained more spectral information reflecting the price of fresh tea leaves, and the established model results will also be better [29].

#### 3.4.2. Principal Component Analysis (PCA)

PCA was applied to extract feature spectral data points and compress the spectral information. The results of the PCA were as follows.

**Figure 7:**
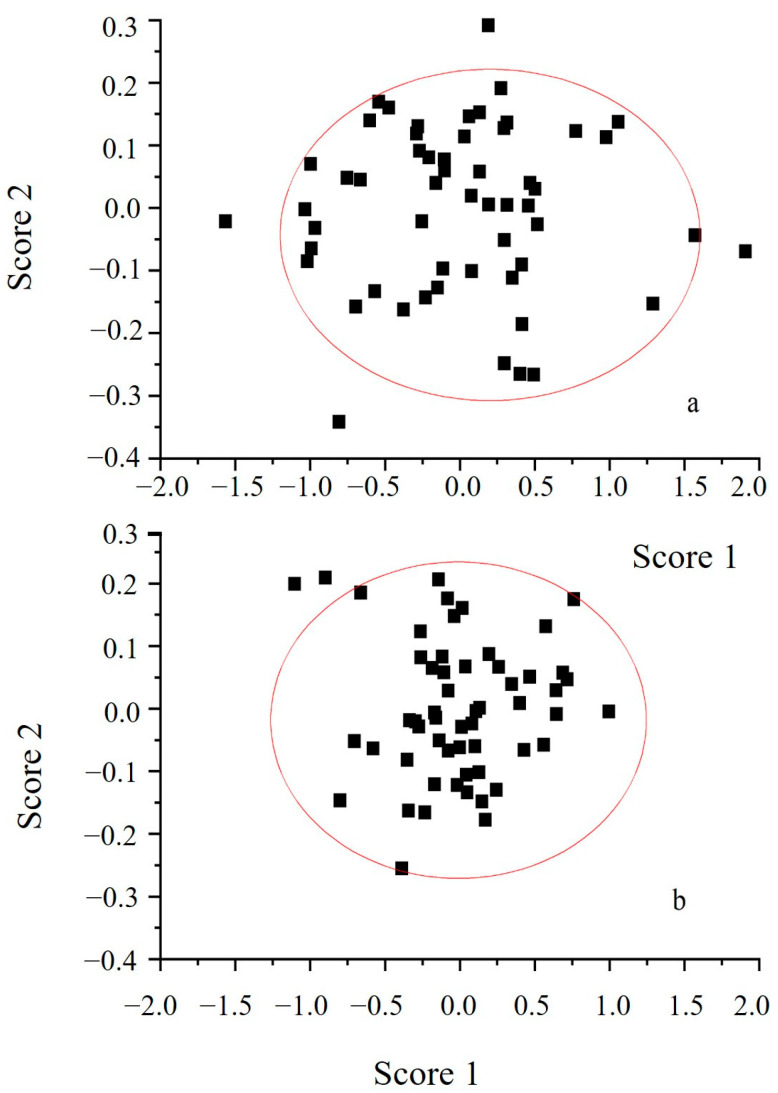
PC1 vs PC2 distribution of different price fresh tea samples. Note: (**a**) was PC1 vs PC2 distribution of DSD 70 data points; (**b**) was PC1 vs PC2 distribution of FSD 33 data points.

From Table 5, it can be concluded that the contribution rates of PC1 for the DSD 70 data points and FSD 33 data points were both higher, reaching 94.828% and 93.101%, respectively. As the increasing of PC2 in the FSD data points has exceeded the increasing of PC2 in the DSD data points (net increase of 2.506%), PC (1–2) was higher than the cumulative contribution rate of the DSD data points. However, the cumulative contribution rates of the first three PCs were very close, at 99.856% and 99.852%, respectively, and so extremely close to 100%. According to the PCA principle [30], the first three PCs can fully represent the extracted spectral data point information, further proving the powerful information extraction ability of the genetic algorithm. From Figure 7, it can be concluded that within the same spatial range, the vast majority of the samples in Figure 7a were distributed within the ranges of −1.0 < score1 < 1.5 and −0.3 < score2 < 0.2, while the vast majority of the samples in Figure 7b were distributed within the ranges of −1.0 < score1 < 1.0 and −0.25 < score2 < 0.2. The distribution space of the samples in Figure 7b was smaller, and the clustering effect was more prominent. This will lay a good foundation for the establishment of the BP-ANN model in the next step.

#### 3.4.3. BP-ANN Model

According to the PCA results, the first three PCs were as input values and the prices were as output values, while the BP-ANN method was used to establish the NIRS models using DSD data points and FSD data points, respectively. During the process of establishing the model, the number of hidden layers was continuously adjusted to obtain the best prediction model. After repeated adjustments, a three-layer BP-ANN prediction model with a 3-5-1 structure was finally obtained using three different transfer functions. This study compared the price NIRS models of three transfer functions, namely the linear [−1, 1] function, logistic function, and tanh function, and the results of the BP-ANN models were as follows.

Table 6 shows that the R_c_^2^ and RMSECV of the calibration price NIRS model established by the linear [−1, 1] transfer function were 0.845 and 11.164 RMB/kg, respectively. When 14 prediction samples were used to verify the robustness, R_p_^2^ and RMSEP were 0.812 and 14.014 RMB/kg, respectively. The Rc^2^ and RMSECV of the calibration price NIRS model established by the tanh transfer function were 0.883 and 10.135 RMB/kg, respectively. When the robustness was verified by 14 prediction samples, the R_p_^2^ and RMSEP were 0.857 and 10.875 RMB/kg, respectively. The Rc^2^ and RMSECV of the calibration price NIRS model established by logistic transfer function were 0.912 and 8.436 RMB/kg, respectively. When the robustness was verified by 14 prediction samples, the R_p_^2^ and RMSEP were 0.873 and 10.364 RMB/kg, respectively.

Table 7 shows that the R_c_^2^ and RMSECV of the calibration price NIRS model established by the linear [−1, 1] transfer function were 0.877 and 10.863 RMB/kg, respectively. When 14 prediction samples were used to verify the robustness, the R_p_^2^ and RMSEP were 0.852 and 10.463 RMB/kg, respectively. The Rc^2^ and RMSECV of the calibration price NIRS model established by the tanh transfer function were 0.925 and 7.912 RMB/kg, respectively. When the robustness was verified by 14 prediction samples, the R_p_^2^ and RMSEP were 0.905 and 7.923 RMB/kg, respectively. The Rc^2^ and RMSECV of the calibration price NIRS model established by logistic transfer function were 0.989 and 5.825 RMB/kg, respectively. When the robustness was verified by 14 prediction samples, the R_p_^2^ and RMSEP were 0.985 and 6.732 RMB/kg, respectively.

From the BP-ANN model results of the three different transfer functions, the model built by linear [−1, 1] transfer function had the worst results, meaning the smallest R^2^ and the largest error. This may be because fresh tea leaves are the site of photosynthesis in tea trees, which produces a large number of chemical substances inside, resulting in extremely rich and complex NIRS information. Moreover, the main substance inside fresh tea leaves is water, accounting for approximately 75% of the fresh weight of the leaves and occupying two very large absorption peaks (5100 cm^−1^ and 6900 cm^−1^) in the NIRS. Therefore, this NIRS information will be squeezed, covering spectral information related to the purchase price of fresh tea leaves, a simple linear relationship is no longer sufficient to establish a satisfactory NIRS price prediction model. Therefore, the BP-ANN model established using the linear transfer function in this study had the worst results. The tanh transfer function is a hyperbolic tangent and nonlinear transfer function with wide applicability [31], and is a widely used transfer function. Therefore, the modeling results of the tanh transfer functions with nonlinearity were slightly better than those of the linear models. The logistic function is an S-shaped nonlinear function [32], a monotone rising function, with good continuity, and the established model is relatively stable. Therefore, among the BP-ANN models established by the three transfer functions, the model results obtained by the logistic function were the best. The results of the calibration model and the prediction model were relatively close, indicating that the model was relatively stable, and there was no overfitting phenomenon.

From the comparison of the results in Table 6 and Table 7, it can also be found that using the same samples, methods, and transfer functions, the results of the model were different due to the different input data point information. The model results of using 33 spectral data points extracted from FSD were better than those using 70 spectral data points extracted from DSD, proving the superiority of the GA in extracting spectral data points most closely related to fresh tea price. It was also proved the superiority of the BP-ANN algorithm. In this study, the results established by the BP-ANN method were better than those obtained by the linear si-PLS method. For samples with very complex internal material components, such as fresh tea leaves, the nonlinear method was a better method for establishing the purchase price model compared to the si-PLS method. The predicted results of the 14 external samples are shown in Table 8 (R^2^ = 0.987, RMSEP = 6.670 RMB/kg).

## 4. Conclusions

In this study, NIRS technology combined with the si-PLS, GA, PCA, and BP-ANN methods was used to successfully establish a robust NIRS digital prediction model for the purchase price of fresh tea leaves. The main conclusions were as follows.

(1) The QI, seven quality grades, and purchase price of fresh tea samples have shown a linear relationship in pairs, with the R^2^ being greater than 0.99. The QI of the seven grades fresh tea samples all had statistical significance (*p* < 0.05).
(2) The best preprocessing method for the original spectra was the combination method of (SNV+SD); four spectral intervals closely related to fresh tea prices were screened out using the si-PLS method, namely 4377.62 cm^−1^–4751.74 cm^−1^, 4755.63 cm^−1^–5129.75 cm^−1^, 6262.70 cm^−1^–6633.93 cm^−1^, and 7386 cm^−1^–7756.32 cm^−1^.
(3) The GA was applied to accurately extract 70 feature spectral data points and 33 feature spectral data points from DSD and FSD, respectively. The cumulative contribution rates of the first three PCs were 99.856% and 99.852%, respectively; However, the spatial distance of the samples extracted from FSD was smaller, and the clustering effect was more pronounced.
(4) The BP-ANN model of price was constructed with the 3-5-1 structure, and the best results were obtained using the logistic transfer function (R_p_^2^ = 0.985, RMSEP = 6.732 RMB/kg). The model results established by 33 feature spectral data points were slightly better than those of 70 feature spectral data points.

In the process of applying this model in the future, it is necessary to expand the range of fresh tea samples at different prices, add samples at different seasons, and establish a more comprehensive and robust prediction model. Then, the price model will be firstly applied to the Enshi Yulu Nature Reserve, providing technological support for the quality of fresh leaves of Enshi Yulu, in order to accumulate application experience.

In addition to continuously improving the prediction accuracy of the model, the consideration of factors such as local fresh tea leaf varieties is also needed to gradually obtain a more refined prediction model. On this basis, the application of the model will be gradually expanded to the whole Hubei province or even to all tea producing areas in China, providing favorable technological support for improving tea quality and promoting high-quality development in the tea industry.

## Figures and Tables

**Table 1:** Composition of seven grades fresh tea leaves samples (fresh weight ratio/%).

Tenderness	Grade 1	Grade 2	Grade 3	Grade 4	Grade 5	Grade 6	Grade 7
Bud	100	0	0	0	0	0	0
One bud and first leaf	0	100	85	75	25	0	0
One bud and two leaves	0	0	15	25	75	75	25
One bud and three leaves	0	0	0	0	0	25	75

**Table 2:** The t values between QI of seven quality grades fresh tea samples.

Grades	1	2	3	4	5	6	7
1	-	3.25	4.23	6.33	9.12	9.31	11.76
2	3.25	-	3.98	5.85	8.82	8.87	11.65
3	4.23	3.98	-	5.20	8.16	8.05	10.63
4	6.33	5.85	5.20	-	7.52	7.48	9.35
5	9.12	8.82	8.16	7.52	-	6.68	8.26
6	9.31	8.87	8.05	7.48	6.68	-	5.21
7	11.76	11.65	10.63	9.35	8.26	5.21	-

Note: t_0.05_(7) = 2.365; t_0.01_(7) = 3.499 [24].

**Table 3:** Results of si-PLS calibration model selected different spectral regions.

Number of Intervals	PLS Factors	Selected Intervals	RMSECV (RMB/kg)
10	9	[1 4]	27.656
11	10	[1 3 7 12]	27.143
12	10	[5 8 10 12]	25.632
13	8	[6 7 10 11]	22.512
14	9	[1 8 10 12]	19.647
15	9	[1 4 7 10]	17.251
16	8	[2 3 7 10]	15.340
17	9	[1 7 12 14]	15.785
18	9	[2 9 10 14]	16.431
19	9	[4 6 8 12]	16.876
20	7	[3 7 11 13]	17.122
21	10	[3 5 7 14]	17.524
22	9	[10 12 15 18]	17.936
23	9	[1 4 7 8]	18.451
24	9	[1 4 8 11]	18.624
25	9	[4 8 11 19]	18.875

**Table 4:** Feature spectral data points extracted by GA.

Spectral Information	Feature Spectral Data Points (cm^−1^)
DSD (70)	4269.63, 4393.05, 4477.90, 4524.18, 4720.89, 4801.88, 4805.74, 4859.74, 4863.59, 4894.45, 4956.16, 4994.73, 5095.01, 5376.57, 5399.71, 5411.28, 5430.56, 5434.42, 5442.13, 5457.56, 5472.99, 5507.70, 5515.42, 5650.41, 5661.98, 5665.84, 5669.69, 5673.55, 5719.83, 5766.12, 5773.83, 5881.82, 6032.24, 6086.24, 6194.24, 6201.95, 6545.22, 6857.63, 6984.91, 7108.33, 7131.47, 7208.61, 7231.75, 7289.60, 7308.89, 7328.17, 7339.74, 7343.60, 7347.46, 7374.46, 7382.17, 7432.31, 7532.59, 7567.30, 7636.73, 7667.58, 7729.29, 7760.15, 7837.29, 7891.29, 7899.00, 8022.42, 8296.26, 8697.39, 8701.24, 8708.96, 8751.38, 9044.51, 9414.77, 9569.05.
FSD (33)	4474.04, 477.90, 4481.76, 4574.32, 4605.18, 4612.89, 4778.74, 4782.60, 4786.45, 4805.74, 4809.60, 4821.17, 4879.02, 4882.88, 4936.88, 4940.73, 4987.02, 5079.58, 5083.44, 5087.30, 5106.58, 5110.44, 5114.29, 6286.80, 6313.80, 6506.65, 6541.36, 6545.22, 6603.07, 6317.66, 6321.51, 6549.07, 7706.15.

**Table 5:** Cumulative contribution rate of the first six principal components.

PCs	PC1	PC (1–2)	PC (1–3)	PC (1–4)	PC (1–5)	PC (1–6)
Cumulative contribution rate of DSD 70 data points/%	94.828	98.558	99.856	99.934	99.968	99.981
Cumulative contribution rate of FSD 33 data points/%	93.101	99.338	99.852	99.957	99.986	99.992

**Table 6:** The results of BP-ANN model established by DSD data points.

Transfer Functions	Calibration Set		Prediction Set	
	R_c_^2^	RMSECV (RMB/kg)	R_p_^2^	RMSEP (RMB/kg)
linear [−1, 1]	0.845	11.164	0.812	14.014
tanh	0.883	10.135	0.857	10.875
logistic	0.912	8.436	0.873	10.364

**Table 7:** The results of BP-ANN model established by FSD data points.

Transfer Functions	Calibration Set		Prediction Set	
	R_c_^2^	RMSECV (RMB/kg)	R_p_^2^	RMSEP (RMB/kg)
linear [−1, 1]	0.877	10.863	0.852	10.463
tanh	0.925	7.912	0.905	7.923
logistic	0.989	5.825	0.985	6.732

**Table 8:** Prediction results of 14 external samples (RMB/kg).

No.	True Values	Predicted Values	No.	True Values	Predicted Values
1	32.5	25.65	8	125	132.2
2	34	40.1	9	146	154.2
3	65	60.5	10	150	142.65
4	73	79.25	11	180	186.3
5	85	80.25	12	186	180.6
6	93	87.62	13	210	200.4
7	118	110.4	14	213	219.0

## Data Availability

The data used to support the findings of this study can be made available by the corresponding author upon request.
